# Hormonal Changes During Menopause and Their Impact on Bone Health: Insights from Orthopedic and Reproductive Medicine

**DOI:** 10.7759/cureus.93224

**Published:** 2025-09-25

**Authors:** Shahmeen Rasul, Yashar Mashayekhi, Maria Javaid, Sami Merie, Marwah A Khalaf, Talha Ahmed, Muhammad Haris, Imtiaz Mustafa

**Affiliations:** 1 Department of Trauma and Orthopaedics, Royal Free Hospital, London, GBR; 2 Department of Medicine, Leicester University Hospital, Leicester, GBR; 3 Department of Obstetrics and Gynaecology, Hameed Latif Teaching Hospital, Lahore, PAK; 4 Department of Trauma and Orthopaedics, University Hospital Lewisham, London, GBR; 5 Department of Community Health Nursing, College of Nursing, University of Kirkuk, Kirkuk, IRQ; 6 Department of Trauma and Orthopaedics, University Hospitals Dorset, Poole Hospital, Poole, GBR; 7 Department of Internal Medicine, King Edward Medical University, Lahore, PAK; 8 Institute of Molecular Biology and Biotechnology, Center for Research in Molecular Medicine, The University of Lahore, Lahore, PAK

**Keywords:** bone mineral density, estrogen, menopause, orthopedic-endocrine link, osteoporosis

## Abstract

Background

Menopause is characterized by profound hormonal alterations, including a decline in estradiol and progesterone with compensatory rises in follicle-stimulating hormone (FSH) and luteinizing hormone (LH). These changes have been implicated in the pathogenesis of bone loss and osteoporosis, yet their precise contribution in the South Asian population remains underexplored. This study aimed to investigate the relationship between hormonal changes during menopause and their impact on bone health, with integrated perspectives from orthopedic and reproductive medicine.

Methods

A retrospective study was conducted at the University of Lahore, Lahore, Pakistan, utilizing medical records of postmenopausal women aged 45-65 years over a 12-month period. A total of 180 participants with complete hormonal and bone health data were included through purposive sampling. Data collected included demographic characteristics, menopausal duration, lifestyle factors, serum hormone levels (estradiol, progesterone, FSH, LH), biochemical markers (serum calcium, vitamin D, alkaline phosphatase), and bone mineral density (BMD) assessed via dual-energy X-ray absorptiometry (DEXA). Statistical analyses included t-tests, chi-square tests, Pearson’s correlation, and multivariate linear regression, with p < 0.05 considered significant.

Results

Women with menopause ≥5 years exhibited significantly lower estradiol (21.4 ± 8.6 pg/mL vs. 36.8 ± 12.4 pg/mL, p < 0.001) and higher FSH (68.7 ± 12.5 mIU/mL vs. 51.6 ± 9.4 mIU/mL, p < 0.001). Lumbar spine BMD was markedly reduced in this group (0.81 ± 0.13 g/cm² vs. 0.94 ± 0.11 g/cm², p < 0.001), with higher rates of osteopenia (49.1%) and osteoporosis (25.5%). Biochemical markers showed lower calcium and vitamin D, alongside elevated alkaline phosphatase. Correlation analysis demonstrated a positive association between estradiol and BMD (r = 0.46, p < 0.001) and a negative association with FSH (r = -0.39, p < 0.001). Multivariate regression identified estradiol as the strongest positive predictor and FSH as the strongest negative predictor of BMD, independent of age, BMI, and lifestyle factors.

Conclusion

Hormonal shifts during menopause, particularly reduced estradiol and elevated FSH, exert a dominant influence on bone health deterioration, surpassing the effects of age, BMI, and lifestyle. Early identification and targeted interventions addressing endocrine changes may be crucial for mitigating osteoporosis risk in postmenopausal women.

## Introduction

Menopause represents a critical physiological transition in a woman’s life, marked by the decline of ovarian function and a sharp reduction in circulating estrogen levels. This hormonal shift initiates a cascade of systemic changes that extend far beyond reproductive cessation, profoundly influencing bone metabolism, musculoskeletal integrity, and overall health outcomes [1]. Estrogen deficiency has been well documented to accelerate bone resorption while simultaneously impairing bone formation, thereby increasing the risk of osteoporosis and fractures through well-established molecular and cellular mechanisms. Furthermore, reduced estrogen not only decreases bone mineral density (BMD) but also compromises the biomechanical strength of bone tissue, rendering it structurally weaker and more prone to fractures even in the absence of significant trauma [2]. In addition, estrogen loss contributes to declines in muscle strength and balance, heightening the risk of falls and associated orthopedic injuries. These combined factors underscore the growing concern of menopausal bone fragility in the context of an aging global population [3].

The term "menopause" encompasses a spectrum of endocrine and clinical changes that affect not only the gynecological domain but also multiple hormone-responsive tissues. These changes underlie conditions such as vaginal atrophy, pelvic floor dysfunction, and urogenital discomfort, reflecting the broader impact of menstrual cessation and subsequent endocrine imbalance. Beyond localized effects, menopausal hormonal disturbances contribute to systemic health outcomes, playing a role in the pathogenesis of chronic conditions such as cardiovascular disease and metabolic syndrome [4]. Estrogen deficiency has been further linked to altered lipid metabolism, heightened inflammatory activity, and increased oxidative stress, all of which elevate the risk of chronic diseases, including cardiovascular disorders and type 2 diabetes [5,6].

Given these multifaceted effects, the medical consequences of menopausal hormonal changes warrant careful exploration within an interdisciplinary framework that integrates gynecology, orthopedics, and internal medicine. Such an approach is crucial for addressing the broad spectrum of postmenopausal health challenges. The complex interplay between hormonal decline and systemic health emphasizes the need for focused investigation into its specific impact on bone health, where estrogen deficiency plays a particularly pivotal role [7].

Therefore, the rationale of the present study lies in the necessity to clarify how hormonal changes during menopause directly affect BMD and related biochemical parameters, thereby advancing understanding of postmenopausal bone health. The objective of this study was to explore the role of hormonal alterations during menopause and their association with bone health outcomes in postmenopausal women.

## Materials and methods

This retrospective study was conducted at the University of Lahore, a tertiary care hospital in Lahore, Pakistan, over a period of 12 months, from June 2024 to May 2025, utilizing medical records of postmenopausal women, both manually and electronically, who had been evaluated for bone health parameters during routine clinical follow-up and diagnostic investigations. The study aimed to explore the role of hormonal changes occurring during menopause and their impact on bone health.

The study population consisted of postmenopausal women aged between 45 and 65 years. A sample size of 180 participants was calculated using OpenEpi software (Open Source Epidemiologic Statistics for Public Health, The OpenEpi Project, Atlanta, GA) with a 95% confidence interval (CI), a power of 80%, and an anticipated effect size of 0.25 between hormonal fluctuations and BMD changes. Non-probability purposive sampling was employed to include all eligible women who had documented records of both hormonal assays and bone health assessments during the specified period.

Inclusion criteria comprised women with a natural history of menopause for at least one year and those with complete medical records, including serum hormonal profiles (estrogen, progesterone, follicle-stimulating hormone (FSH), and luteinizing hormone (LH)) and bone health assessments (BMD, serum calcium, vitamin D (25-hydroxyvitamin D), and alkaline phosphatase). Exclusion criteria included women with premature or surgically induced menopause, those with chronic systemic diseases such as renal or hepatic impairment, individuals on long-term (more than six months) corticosteroid or hormone replacement therapy, and patients with incomplete or missing clinical data.

Data collection involved reviewing the hospital information management system and retrieving demographic details, reproductive history, duration since menopause, and lifestyle factors such as smoking, physical activity, and dietary calcium intake. Hormonal parameters, including serum estradiol, progesterone, FSH, and LH, were measured in the clinical biochemistry laboratory of the University of Lahore using enzyme-linked immunosorbent assay (ELISA) kits with standardized protocols. Bone health was assessed through dual-energy X-ray absorptiometry (DEXA) scans, which provided BMD values at the lumbar spine and femoral neck. Additionally, biochemical markers such as serum calcium, serum vitamin D, and alkaline phosphatase were obtained from laboratory records to complement radiological findings.

Data were entered and analyzed using the IBM SPSS Statistics software, version 26 (IBM Corp., Armonk, NY). Continuous variables such as hormone levels and BMD values were expressed as mean ± standard deviation, whereas categorical variables such as menopausal duration categories were presented as frequencies and percentages. Normality of distribution was assessed using the Shapiro-Wilk test. Group comparisons between different durations of menopause were carried out using independent sample t-tests or ANOVA, depending on the number of groups. Pearson’s correlation coefficient was applied to explore the association between hormonal levels and BMD. Multiple linear regression analysis was performed to adjust for potential confounders, including age, BMI, and lifestyle factors. A p-value of less than 0.05 was considered statistically significant.

All data were retrieved from hospital medical records. Patient confidentiality was strictly maintained, and no personal identifiers were included in the study. As this was retrospective research of existing records, no direct patient interaction was involved, and informed consent was not required. Formal ethical approval was not obtained; however, the study adhered to institutional standards for the use of anonymized retrospective data.

## Results

A total of 180 postmenopausal women were included in the study, with a mean age of 55.8 ± 5.7 years and a mean menopausal duration of 7.4 ± 3.8 years. The baseline demographic and lifestyle characteristics of the study group are summarized in Table 1. The majority of participants had had menopause for more than five years (61.1%), while nearly one-quarter were smokers, and around 42% reported being physically active. Notably, about 41.6% of the women had low dietary calcium intake, highlighting a potential nutritional risk factor for impaired bone health (Table 1).

**Table 1:** Baseline demographic and lifestyle characteristics of the study participants (n = 180) Data are presented as mean ± standard deviation or frequency (%). Descriptive statistics only.

Variable	Mean ± SD / n (%)
Age (years)	55.8 ± 5.7
BMI (kg/m²)	27.3 ± 3.6
Duration since menopause (years)	7.4 ± 3.8
Menopause < 5 years	70 (38.9%)
Menopause ≥ 5 years	110 (61.1%)
Smokers	42 (23.3%)
Physically active	76 (42.2%)
Low dietary calcium intake	75 (41.6%)

Hormonal profile

Estradiol and progesterone levels were significantly lower among women with ≥5 years since menopause, whereas gonadotropins (FSH and LH) were significantly elevated, consistent with the endocrine changes of postmenopausal physiology. These differences suggest a progressive worsening of hormonal imbalance with increasing menopausal duration, potentially aggravating bone health decline (Table 2).

**Table 2:** Hormonal profile of the study participants by duration of menopause Independent sample t-test was used. Effect size reported as Cohen’s d. FSH: follicle-stimulating hormone; LH: luteinizing hormone

Hormonal Parameter (Normal Range)	< 5 years (n=70) Mean ± SD	≥ 5 years (n=110) Mean ± SD	Test statistic	p-value
Estradiol (15–350 pg/mL)	36.8 ± 12.4	21.4 ± 8.6	t(178) = 9.03, d = 0.89	<0.001
Progesterone (0.1–0.8 ng/mL postmenopausal)	0.82 ± 0.21	0.54 ± 0.17	t(178) = 9.05, d = 0.91	<0.001
FSH (25–135 mIU/mL postmenopausal)	51.6 ± 9.4	68.7 ± 12.5	t(178) = -9.68, d = 0.97	<0.001
LH (15–62 mIU/mL postmenopausal)	26.1 ± 7.3	34.2 ± 8.8	t(178) = -6.68, d = 0.74	<0.001

BMD

BMD was significantly lower in women with ≥5 years since menopause at both the lumbar spine and femoral neck. Moreover, the prevalence of osteopenia and osteoporosis was higher in this group, with chi-square tests confirming statistically significant differences. These findings indicate a cumulative effect of menopausal duration on the deterioration of bone health and emphasize the vulnerability of women with prolonged estrogen deficiency (Table 3).

**Table 3:** Bone mineral density (BMD) and prevalence of bone health conditions Independent sample t-tests for continuous BMD measures; chi-square tests for categorical prevalence. Effect sizes are reported as Cohen’s d (continuous) and phi coefficient (categorical).

Site/Condition (Normal Range)	< 5 years (n=70) Mean ± SD / n (%)	≥ 5 years (n=110) Mean ± SD / n (%)	Test statistic	p-value
Lumbar spine BMD (≥1.0 g/cm² normal)	0.94 ± 0.11	0.81 ± 0.13	t(178) = 6.86, d = 0.82	<0.001
Femoral neck BMD (≥0.9 g/cm² normal)	0.87 ± 0.09	0.75 ± 0.12	t(178) = 7.31, d = 0.86	<0.001
Osteopenia (T-score: -1.0 to -2.5)	22 (31.4%)	54 (49.1%)	χ²(1) = 5.62, φ = 0.18	0.018
Osteoporosis (T-score: ≤ -2.5)	6 (8.6%)	28 (25.5%)	χ²(1) = 7.32, φ = 0.20	0.007

Biochemical markers of bone health

Biochemical markers reflected consistent patterns with BMD findings. Women with a menopausal duration ≥5 years had significantly lower serum calcium and vitamin D levels, alongside higher alkaline phosphatase, indicating enhanced bone turnover and metabolic stress. These biochemical alterations further substantiate the detrimental impact of long-standing estrogen deficiency on skeletal integrity (Table 4).

**Table 4:** Biochemical markers of bone health by duration of menopause Independent sample t-tests used. Effect size are reported as Cohen’s d.

Parameter (Normal Range)	< 5 years (n=70) Mean ± SD	≥ 5 years (n=110) Mean ± SD	Test Statistic	p-value
Serum calcium (8.5–10.5 mg/dL)	9.1 ± 0.6	8.5 ± 0.7	t(178) = 6.16, d = 0.72	<0.001
Vitamin D, 25(OH)D (20–50 ng/mL sufficient)	27.8 ± 6.3	20.4 ± 5.9	t(178) = 7.83, d = 0.83	<0.001
Alkaline phosphatase (40–120 U/L)	86.5 ± 19.7	112.4 ± 22.3	t(178) = -8.10, d = 0.84	<0.001

Correlation between hormones and BMD

Pearson’s correlation analysis showed a strong positive association between estradiol and BMD at both sites, indicating the protective role of estrogen. Conversely, FSH and LH were negatively correlated with BMD, highlighting their detrimental influence on bone health. Progesterone showed only a weak but statistically significant correlation. These findings reaffirm the central role of estrogen decline and gonadotropin rise in menopausal bone deterioration (Table 5).

**Table 5:** Correlation between hormonal levels and bone mineral density (BMD) Pearson correlation coefficients (r) are reported with corresponding p-values. FSH: follicle-stimulating hormone; LH: luteinizing hormone

Parameter	Lumbar Spine BMD (r, p)	Femoral Neck BMD (r, p)
Estradiol	0.46, p < 0.001	0.41, p < 0.001
Progesterone	0.22, p = 0.005	0.19, p = 0.011
FSH	0.39, p < 0.001	-0.34, p < 0.001
LH	-0.31, p < 0.001	-0.29, p = 0.002

Multivariate regression analysis

Multivariate regression revealed estradiol as the strongest independent positive predictor of BMD, whereas FSH emerged as a significant negative predictor, even after adjusting for age, BMI, smoking, and calcium intake. Age itself remained an independent negative predictor, whereas BMI showed no significant independent effect. These results suggest that hormonal shifts, particularly reduced estradiol and elevated FSH, are the most influential determinants of postmenopausal bone health (Table 6).

**Table 6:** Multivariate regression analysis for predictors of bone mineral density (BMD) Multivariate linear regression analysis performed. Standardized regression coefficients (β), standard errors (SE), and p-values are reported. FSH: follicle-stimulating hormone; LH: luteinizing hormone

Predictor	Lumbar Spine BMD (β, SE) , p	Femoral Neck BMD (β, SE), p
Estradiol	0.38 (0.07), p < 0.001	0.34 (0.08), p < 0.001
Progesterone	0.11 (0.06), p = 0.074	0.09 (0.06), p = 0.112
FSH	-0.29 (0.07), p < 0.001	-0.24 (0.08), p = 0.002
LH	-0.13 (0.07), p = 0.052	-0.11 (0.07), p = 0.067
Age	-0.19 (0.07), p = 0.007	-0.16 (0.06), p = 0.013
BMI	0.09 (0.06), p = 0.118	0.08 (0.06), p = 0.132

## Discussion

The present study investigated the role of hormonal changes during menopause and their impact on bone health among postmenopausal women. Our findings demonstrated that the duration of menopause was significantly associated with adverse alterations in hormonal levels, BMD, and biochemical markers of bone metabolism, underscoring the critical role of estrogen deficiency in the pathophysiology of postmenopausal osteoporosis.

In terms of baseline demographics, the mean age of participants was 55.8 years, with a mean menopausal duration of 7.4 years. The prevalence of smoking, sedentary lifestyle, and low dietary calcium intake in our sample was consistent with regional studies highlighting similar lifestyle risk factors for osteoporosis in South Asian populations [8, 9]. The relatively high BMI observed in our cohort may provide partial protective effects against bone loss, as suggested in prior literature; however, obesity-related inflammation may offset this benefit in the long term [10].

The hormonal profile analysis confirmed profound reductions in estradiol and progesterone, alongside significant elevations in FSH and LH, particularly among women more than five years into menopause. These results align with the well-established endocrinological changes characterizing menopause, where depletion of ovarian reserve leads to diminished estrogen and progesterone production with compensatory gonadotropin elevation [11]. Similar hormonal patterns have been reported by Yureneva et al., who demonstrated that estradiol declines rapidly during the menopausal transition and remains suppressed thereafter, while FSH and LH continue to rise [12]. Our study strengthens these observations by directly linking the hormonal profile with bone health outcomes.

BMD results revealed significant reductions at both the lumbar spine and femoral neck in women with longer menopausal duration. The prevalence of osteopenia (49.1%) and osteoporosis (25.5%) in women beyond five years of menopause was markedly higher than in those with a shorter menopausal duration. These findings are in line with large epidemiological surveys such as the Women’s Health Initiative (WHI) and studies by Suvvari et al., which reported that the risk of osteoporosis and fragility fractures increases substantially with menopausal duration due to progressive estrogen deficiency [13]. Interestingly, our observed rates of osteoporosis were slightly higher than those reported in Western cohorts, which may reflect ethnic differences in peak bone mass and dietary calcium/vitamin D intake, as highlighted in prior studies from Asian populations [14,15].

Biochemical markers of bone health in our study provided further insight into the mechanisms of postmenopausal bone loss. Women with longer menopausal duration exhibited significantly lower serum calcium and vitamin D levels, alongside higher alkaline phosphatase. Reduced vitamin D in this group likely reflects inadequate sun exposure and dietary intake, compounded by aging-related decline in cutaneous synthesis, as documented by Lips and colleagues [16]. Elevated alkaline phosphatase in our cohort is consistent with enhanced bone turnover in estrogen-deficient states, corroborating findings by Fine et al., who linked increased bone turnover markers to accelerated postmenopausal bone loss [17].

Correlation analysis revealed that estradiol was positively associated with BMD at both the lumbar spine and femoral neck, while FSH and LH were inversely correlated. These findings are in agreement with the mechanistic role of estrogen in promoting osteoblastic activity and suppressing osteoclastic resorption, while elevated gonadotropins indirectly exacerbate bone resorption [18]. Progesterone showed only a modest positive correlation with BMD, reflecting its limited direct skeletal role compared to estradiol. Similar patterns have been reported in longitudinal studies, emphasizing estradiol as the most consistent hormonal determinant of postmenopausal BMD [19].

Multivariate regression confirmed estradiol as the strongest independent predictor of BMD, even after adjustment for age, BMI, smoking, and dietary calcium. Conversely, FSH emerged as an independent negative predictor. These findings resonate with the work of Sowers et al. and Karim et al., who demonstrated that estradiol levels are robust predictors of postmenopausal bone density and fracture risk, whereas gonadotropin levels contribute independently but to a lesser degree [20, 21]. Age was also found to be a significant predictor, consistent with the natural decline in bone mass with aging beyond hormonal changes. BMI, although positively associated with BMD, did not reach statistical significance, which may reflect the modest sample size and the confounding influence of adiposity-related inflammation [22].

Overall, our findings highlight that menopausal hormonal changes exert a profound influence on bone health, mediated primarily through estradiol deficiency and reflected in both densitometric and biochemical markers. These results corroborate a wealth of international literature while also emphasizing population-specific factors such as lifestyle, diet, and baseline bone mass. Importantly, they underscore the clinical need for early identification of women at higher risk of osteoporosis, particularly those with prolonged menopausal duration, low vitamin D, and unfavorable hormonal profiles.

The strengths of this study include its comprehensive evaluation of hormonal, biochemical, and densitometric parameters within the same cohort, as well as robust statistical adjustment for key confounders. However, certain limitations warrant consideration, including its retrospective design, single-center design, reliance on single-point biochemical measurements, and absence of longitudinal fracture data. Future prospective studies with larger and more diverse populations are needed to further elucidate the temporal relationships between hormonal decline and skeletal outcomes.

## Conclusions

Our study demonstrates that menopausal hormonal alterations, particularly estradiol decline and gonadotropin elevation, are strongly associated with reductions in BMD, alterations in biochemical markers of bone metabolism, and increased prevalence of osteoporosis. These findings reinforce the critical role of hormonal assessment and bone health monitoring in postmenopausal women, and they provide evidence to support targeted preventive strategies, including lifestyle modification, vitamin D optimization, and timely therapeutic intervention.
